# Severe Chikungunya infection in Northern Mozambique: a case report

**DOI:** 10.1186/s13104-017-2417-z

**Published:** 2017-02-08

**Authors:** Mussa Manuel Aly, Sadia Ali, Argentina Felisbela Muianga, Vanessa Monteiro, Jorge Galano Gallego, Jacqueline Weyer, Kerstin I. Falk, Janusz Tadeusz Paweska, Julie Cliff, Eduardo Samo Gudo

**Affiliations:** 1 Pemba Operational Research Unit, Ministry of Health, Pemba, Mozambique; 2 National Institute of Health, Ministry of Health, Av Eduardo Mondlane 1008, Ministry of Health Main Building, 2nd Floor, PO Box 264, Maputo, Mozambique; 3 Cabo Delgado Provincial Health Directorate, Pemba, Mozambique; 4 Pemba Provincial Hospital, Pemba, Mozambique; 5 Centre for Emerging and Zoonotic Diseases, National Institute for Communicable Disease, National Health Laboratory Service, Sandringham, South Africa; 6 The Public Health Agency of Sweden, Stockholm, Sweden; 7 Department of Microbiology, Tumor and Cell Biology, Karolinska Institutet, Stockholm, Sweden; 8 Faculty of Health Sciences, School of Pathology, University of Witwatersrand, Johannesburg, South Africa; 9 Community Health Department, Faculty of Medicine, Eduardo Mondlane University, Maputo, Mozambique

**Keywords:** Case reports, Chikungunya, Arboviruses, Fever, Mozambique

## Abstract

**Background:**

Although Chikungunya virus has rapidly expanded to several countries in sub-Saharan Africa, little attention has been paid to its control and management. Until recently, Chikungunya has been regarded as a benign and self-limiting disease. In this report we describe the first case of severe Chikungunya disease in an adult patient in Pemba, Mozambique.

**Case presentation:**

A previously healthy 40 year old male of Makonde ethnicity with no known past medical history and resident in Pemba for the past 11 years presented with a severe febrile illness. Despite administration of broad spectrum intravenous antibiotics the patient rapidly deteriorated and became comatose while developing anaemia, thrombocytopenia and later, melaena. Laboratory testing revealed IgM antibodies against Chikungunya virus. Malaria tests were consistently negative.

**Conclusions:**

This report suggests that Chikungunya might cause unsuspected severe disease in febrile patients in Mozambique and provides insights for the improvement of national protocols for management of febrile patients in Mozambique. We recommend that clinicians should consider Chikungunya in the differential diagnosis of febrile illness in locations where *Aedes aegypti* mosquitos are abundant.

## Background

Chikungunya virus (CHIKV), an arthropod borne virus transmitted to humans primarily by the *Aedes aegypti* mosquito, has recently emerged as a serious public health threat [1, 2]. This virus has spread at an alarming rate in the last decade, causing several outbreaks in sub-Saharan Africa [3–6], Asia [7–9], Europe [10, 11] and the Americas [12–15].

Until recently, CHIKV infection has been regarded as a benign and self-limiting disease, characterized by the presence of arthralgia, fever and rash [16–18]. But the occurrence of a massive CHIKV outbreak on Reunion Island in 2005–2006, with an estimated 266,000 cases including 254 deaths, challenged the previous belief that severe disease is a rare outcome of CHIKV infection [19–23]. Although arthralgia is considered the hallmark of the disease, the full spectrum of clinical manifestations may be wider because CHIKV can also affect hepatic, renal, neurological and cardiovascular functions [16, 20, 24–26]. Severe CHIKV disease has been rarely reported in sub-Saharan Africa, most likely due to lack of awareness and lack of laboratory capacity (4, 27, 32). Severe cases are probably under-diagnosed in the sub-Saharan African region, including Mozambique. For instance, a recent study on febrile patients in Mozambique, reported nine cases of acute CHIKV infection, all with benign and self-limiting disease [27]. Furthermore, studies from Asia and the Americas have shown that under-reporting of severe CHIKV disease is common in affected areas [22, 23]. Understanding the clinical spectrum of CHIKV disease could increase the clinical awareness of the disease in Mozambique and improve algorithms for clinical and laboratory management of febrile illness. The detection of a case of Chikungunya may also increase institutional and government awareness of the need to develop epidemiological tools to improve reporting and analysis of routine data in order to allow periodic disclosure of information and promote the exchange of experiences between different provinces and municipalities. Here, we describe the first case of CHIKV disease with a severe clinical course from an adult patient in Pemba, situated in northern Mozambique. Of note, Pemba was hit by an outbreak of Dengue during the same period [28].

## Case presentation

A previously healthy 40 year old male of Makonde ethnicity, resident in Pemba for the past 11 years with no other known past medical history except controlled hypertension was admitted to Pemba Provincial Hospital on April 16th, 2014, complaining of 2 days of fever, chills, malaise, general weakness and prostration, with no headache, arthralgia or myalgia.

Upon admission, the patient was immediately tested for malaria as per the national algorithm using a Rapid Diagnostic Test (Malaria Ag P.f, SD BIOLIN, Alere) and found to be negative (see Table 1). Physical examination showed an axillary temperature of 38.2 °C, blood pressure of 110/75 mmHg and an undifferentiated skin rash. Neither skin nor mucosal haemorrhages nor oedema were present. Table 1 shows the results of all laboratory tests. A full blood count and clinical chemistry measurements showed leukocytosis [white blood count (WBC) = 15.7 × 10^3^ cell/mm^3^], lymphocytosis [Lymphocytes (Ly) = 6.9 × 10^3^ cell/mm^3^], severe thrombocytopenia [Platelets (PLT) = 33 × 10^3^ cell/mm^3^), anaemia [Haemoglobin (Hgb) = 9.8 gr/dL] and an elevated level of Alanine Amino Transferase (ALT = 245 U/L). Blood levels of glucose and urea were slightly elevated. The laboratory did not have the capacity to perform a blood culture. Despite treatment with broad spectrum antibiotics, the patient’s condition progressively worsened over the following 2 days, with a continuing high fever and the development of headache, dizziness, symmetrical polyarthralgia and melaena. On day two after admission the Hgb had dropped to 6.2 g/dL and the platelet count had increased slightly to 56 × 10^3^ cell/mm^3^. On day three the patient worsened and he was admitted to the intensive care unit where he received 3 units of whole blood. Deterioration continued on day four and the patient became comatose. Neither an electroencephalogram (EEG) nor brain scan were performed, as these technologies are not available in most hospitals in Mozambique, including Pemba Provincial Hospital. Malaria tests performed on days one, three and four were all negative. Results of a urine test performed on day four found that cyto-chemical parameters were within the normal range (pH = 6.8, specific density = 1025, nitrite, protein, ketone, bilirubin and blood all negative, leukocytes = 4/μL and red blood cells = 2/μL). Urine culture was negative. During hospitalization the patient was treated with the following antibiotics: ceftriaxone, ciprofloxacin and ampicillin.

**Table 1:** Patient’s laboratory results

Parameter	Day 1	Day 2	Day 3	Day 4	Day 7	Day 12	Day 19	Day 44
White blood cells (10^3^ cell/mm^3^)	15.7	26.7	28.8	21.7	16.4	5.8	9.2	8.3
Lymphocytes (10^3^ cell/mm^3^)	6.9	13.4	11.0	10.3	9.0	2.9	1.89	3.1
Lymphocytes (%)	43.9	50.1	38.3	47.6	59.0	49.7	20.5	37.3
Neutrophils (10^3^ cell/mm^3^)	–	–	13.2	8	–	2.05	6.5	4.4
Neutrophils (%)	–	–	45.9	36.8	–	35.2	70.8	53.5
Haemoglobin (g/dL)	9.8	6.2	6.1	6.6	8.8	10.0	10.4	11.9
Red blood cells (10^6^ cell/mm^3^)	4.4	2.61	2.47	2.85	3.67	4.14	4.25	4.83
Haematocrit (%)	33	19.2	19.7	21	29.9	33.3	34.2	38.0
Mean cell volume (fL)	74.3	75.6	79.8	76.8	81.5	80.4	80.5	78.7
Platelets (10^3^ cell/mm^3^)	33	56	164	222	182	420	337	396
Glucose (mmol/L)	9.11	nt	nt	nt	4.06	5.0	nt	nt
AST (U/L)	0.7	nt	nt	nt	2.54	129.8	nt	nt
ALT (U/L)	245	nt	nt	nt	155	154	nt	
ALP (g/L)	nt	nt	nt	nt	nt	nt	nt	118
Direct bilirubin (μmol/L)	nt	nt	nt	nt	5.4	2.64	nt	1.35
Total bilirubin (μmol/L)	nt	nt	nt	nt	5.7	10.39	nt	4.40
Creatinine (μmol/L)	89.9	nt	nt	nt	83.02	85.2	nt	81.3
Urea (mmol/L)	14.2	nt	nt	nt	nt	3.3	nt	5.3
Uric acid (μmol/L)	nt	nt	nt	nt	374.9	324.1	nt	nt
Sodium (mmol/L)	nt	nt	nt	nt	nt	nt	nt	139.4
Potassium (mmol/L)	nt	nt	nt	nt	nt	nt	nt	4.3
Albumin (g/L)	nt	nt	nt	nt	nt	nt	nt	41.9
Plasmodium falciparum	Neg	nt	Neg	Neg	nt	nt	nt	Neg

*AST* aspartate aminotransferase, *ALT* alanine aminotransferase, *ALP* alkaline phosphatase, *nt* not tested, *Neg* negative

The broad spectrum antibiotic therapy was continued since leukocytosis and lymphocytosis persisted. On day five the patient began to recover and woke from coma. Blood pressure remained controlled throughout the admission.

The patient’s clinical presentation improved progressively and on day seven the Hgb level was 8.8 g/dL, the leukocyte count and ALT level had dropped to 16.4 × 10^3^ cell/mm^3^ and 155 U/L, respectively, and the platelet count had increased to 182 × 10^3^ cell/mm^3^. He was discharged from hospital on the same day with intermittent low grade melaena, but with neither epigastric pain nor hematemesis. On day 12 he returned for a follow-up visit and presented a normal leukocyte, lymphocyte and platelet count, the Hgb had increased to 10 g/dL, but the ALT was still slightly elevated at 155 U/L. The patient reported continuing intermittent low grade melaena.

On day 40, the patient complained of malaise and intermittent low grade melaena and was transferred to a private hospital in Maputo City, the capital of the country, where he was followed up as an outpatient. On day 44 various tests were performed, including full blood count, chest X ray and clinical chemistry, but all were normal (see Table 1). Upper gastrointestinal endoscopy showed no evident cause of melaena: the oesophagus, stomach and duodenum were normal. Colonoscopy was also normal.

Blood samples were sent to the Centre for Emerging and Zoonotic Diseases of the National Institute of Communicable Disease in South Africa for Dengue testing. No serological evidence of Dengue was found using an in house developed haemagglutination inhibition test (HAI) and a commercial Dengue IgM capture Enzyme Linked Immunoabsorbent Assay (ELISA, Panbio, Australia). In addition, the sample was submitted to a wider arboviral screen, and was positive for CHIKV using the CHIKV HAI and commercial IgM ELISA for CHIKV (Eurommune, Germany). These findings clearly point to a recent infection by CHIKV. The patient was treated with antacids and antibiotics. At the follow up visit on day 60 he had completely recovered and was without melaena or any other remaining sequelae.

## Discussion

Despite the recent expansion of CHIKV globally, very little attention has been paid to its management and control in sub-Saharan Africa, as most of the reported cases in the region are described as self-limiting with a benign outcome [4, 6, 29]. In this manuscript we describe a case in northern Mozambique of recent severe CHIKV disease complicated by upper digestive haemorrhage, severe anaemia, thrombocytopenia, and rapid progression to coma.

Malaria was excluded, as test results were consistently negative. Bacteraemia was initially suspected as the patient had a leukocytosis, but the worsening of the disease irrespective of the intensive administration of broad spectrum antibiotics suggested a nonbacterial aetiology. The inability to perform haemoculture due to lack of laboratory capacity is a limitation of this study. Additionally, the presence of thrombocytopenia, upper digestive haemorrhage and, more importantly, the presence of symmetrical polyarthralgia and skin rash was suggestive of mosquito borne viral disease. The concomitant occurrence of a Dengue outbreak in Pemba during the same period also represents a strong argument in favour of infection by a mosquito borne virus [28, 30].

Other causes of haemorrhage that might occur in this region were considered as part of the differential diagnosis. Among the potential causes, leptospirosis and rickettsiosis were considered unlikely, as the patient did not report any exposure to an environment where these pathogens might occur. Also, the patient did not improve despite administration of broad spectrum antibiotics known to be effective against these diseases. Leptospirosis was also excluded because the signs and symptoms of severe disease were absent and as such, renal function was normal throughout the disease, jaundice was absent, the bilirubin level was normal, and no respiratory distress was observed. Hantavirus was also excluded as renal and respiratory dysfunction were absent and also because the occupational and residence history of the patient did not suggest exposure to rodents or places with poor sanitation. Strong arguments in favour of CHIKV are the presence of symmetrical polyarthralgia, skin rash and thrombocytopenia as shown in previous reports during CHIKV outbreaks in other countries [16, 31].

As in this patient, elevated white cell counts have previously been noted in case reports of patients with severe CHIKV disease [32–35]. A recent study by Rolle et al. [36] to assess predictive factors for severe forms of CHIKV disease found higher leukocyte counts in patients with severe disease. The leukocyte count may also be used to distinguish CHIKV from Dengue. Laoprasopwattana et al. [34] proposed that a white cell count of >5000 cells/mm^3^ could help in the differential diagnosis of CHIKV and Dengue infection. Lee et al. in a study conducted in Singapore also demonstrated that the presence of elevated counts of leukocytes was a strong predictor variable to differentiate CHIKV infected patients from Dengue infected patients [37]. Indeed Rolle et al. [36] in Guadeloupe, Kee et al. [38] in Singapore and Chua et al. [39] in Malaysia also found that patients with severe CHIKV infection had a higher leukocyte count and their urine and/or blood culture results were negative. If presence of leukocytosis represents a predictive variable for severity, a consequence of CHIKV infection, or a mere coincidence still deserves a comprehensive investigation, especially in view of the growing number of reported cases of CHIKV infected patients with leukocytosis. Other studies have found contradictory data, with CHIKV infected patients presenting equivalent [40] or lower leukocyte counts [16, 17] compared to uninfected patients. Nonetheless, during the literature review we found at least three authors arguing that CHIKV infection may lead to sepsis, which might explain the presence of leukocytosis [33, 36, 41].

Haemorrhagic signs, although rare, can occur in CHIKV infected patients, as shown in several reports [31, 40, 42]. They were reported in 6.4% of CHIKV infected patients in Reunion Island [31] and 2.2% of patients in Gabon [40]. Other causes of upper digestive haemorrhage were excluded by intestinal endoscopy and colonoscopy.

Brain involvement and encephalitis have been repeatedly reported in patient series, mostly in India [43–46] and Reunion Island [20, 47, 48], showing that neurological involvement is not a rare event during CHIKV infection. Encephalitis with alteration of consciousness is considered the most common neurological manifestation of CHIKV infection [26, 41, 43, 49] and is an important cause of death [45, 48]. The unavailability of an EEG and brain scan is a limitation, although Rajapakse et al. [26], in a review of atypical manifestations of CHIKV infection, suggest that, from the limited evidence available, EEG changes appear to be non-specific. Knowledge of the value of neuroimaging is limited.

Of note, the literature reviews conducted by Rajapakse et al. [26] and Arpino et al. [49], as well as a series of case reports by Taraphdar et al. [46], described the development of neurological manifestation a few days after disease onset, with recovery a few days later, corroborating the findings in our patient. The mechanisms leading to encephalitis are not well understood, but previous authors have speculated that persistence of the virus or an inappropriate immune response might be considered [43].

Renal and liver function tests were mostly within normal ranges, except for ALT, which was persistently elevated, and urea, which was slightly elevated on admission. Elevated levels of ALT have also been noted in previous case reports of CHIKV [16, 17, 32, 40].

Blood glucose was also slightly elevated on admission.

Based on this report, we believe that in Mozambique and other sub-Saharan countries, severe cases of CHIKV infection may be more frequent than previously thought, because most of severe cases are misdiagnosed as malaria or other diseases. Our assumptions are strongly corroborated by findings of two studies conducted in India and Colombia, which showed that deaths were rarely reported during CHIKV outbreaks, although statistical projections demonstrated that in fact, dozens or hundreds of deaths had occurred during the same period [22, 23]. In addition, an increased frequency of severe CHIKV disease has been reported in recent outbreaks in Reunion Island and India [20, 23, 26], suggesting that the benign nature of CHIKV disease should be reconsidered. As such, we argue that severe cases of CHIKV infection are likely more frequent in Mozambique and other sub-Saharan countries than previously suspected.

There was a delay to test and confirm the presence of IgM antibodies against CHIKV, as laboratory capacity was unavailable in country. The sample was, therefore, tested in a regional laboratory in South Africa. The lack of laboratory capacity to support clinical suspicion is a serious challenge to the proper management of febrile illness in Mozambique. Other reasons that are known to contribute to under diagnosis of CHIKV are, (1) similar clinical presentation to malaria and Dengue and (2) lack of clinical awareness of CHIKV, as data on the epidemiology of CHIKV in Mozambique and other sub-Saharan African countries are scarce [50, 51].

## Conclusions

This report suggests that CHIKV may cause unsuspected severe disease in febrile patients in Mozambique. Most likely, these cases are misdiagnosed and treated with anti-malarial drugs or antibiotics. This case report provides insights for the improvement of national protocols for management of febrile patients in Mozambique and we recommend that clinicians should consider CHIKV in the differential diagnosis of febrile illness in locations where *A. aegypti* mosquito is abundant.

## Abbreviations

ALP: alkaline phosphatase
ALT: alanine amino transferase
AST: aspartate aminotransferase
CHIKV: Chikungunya virus
EEG: electroencephalogram
ELISA: enzyme linked immunoabsorbent assay
HAI: haemagglutination inhibition test
Hgb: haemoglobin
Ly: lymphocytes
PLT: platelets
WBC: white blood count
